# Masking Ability of CAD‐CAM Materials at Different Thicknesses: A Study on Color, Translucency, and Infinite Optical Thickness

**DOI:** 10.1111/jerd.13490

**Published:** 2025-05-27

**Authors:** Bruno Arruda Mascaro, Maria Tejada‐Casado, Renata Garcia Fonseca, Juan C. Cardona, José Maurício dos Santos Nunes Reis, María M. Pérez

**Affiliations:** ^1^ Department of Dental Materials and Prosthodontics São Paulo State University (UNESP), School of Dentistry São Paulo Brazil; ^2^ Department of Optics, Faculty of Science, Instituto de Investigación Biosanitaria ibs.GRANADA University of Granada Granada Spain

**Keywords:** color, dental materials, infinite optical thickness, masking ability, thickness color

## Abstract

**Objective:**

To evaluate the masking ability of different thicknesses of CAD‐CAM resin‐matrix and feldspar porcelain materials.

**Materials and Methods:**

Specimens (Ø7.5 mm, thickness 0.5, 1.0, and 1.5 mm, *n* = 3/material) were fabricated from Lava Ultimate‐LU, Grandio Blocs‐GB, VITA Enamic‐VE, and VITA Mark II‐VM. Backgrounds were obtained from nine composite die shades (ND1‐ND9, IPS Natural Die Material). Spectral reflectance was measured for samples against a black background and each ND background. CIEDE2000 color differences (Δ*E*_00_), relative translucency parameter differences (ΔRTP_00_), and infinite optical thickness (*X*_∞_) were calculated. Δ*E*_00_ data among materials were analyzed using Kruskal–Wallis and Mann–Whitney tests (*α* = 0.05). Δ*E*_00_ between thicknesses and ΔRTP_00_ data were compared to perceptible and acceptable thresholds. Root mean square error (RMSE) and goodness‐of‐fit (GFC) were used as performance behavior of *X*_∞_.

**Results:**

VM exhibited the lowest Δ*E*_00_ (*p <* 0.001) and ΔRTP_00_ values across all tested conditions. Overall, LU and GB did not differ for 0.5 and 1.0 mm. Regardless of the background, when comparing 1.0–1.5 mm, acceptable Δ*E*_00_ were observed, with only VM showing imperceptible values. Good spectral match and good comparative values of *X*_∞_ were found for LU‐GB.

**Conclusion:**

The masking effect depended on material and thickness, with VM and thicker samples demonstrating superior results. In general, the spectral behavior and values of infinite optical thickness differed among materials.

**Clinical Significance:**

The masking ability of high‐translucency CAD‐CAM materials was influenced by composition, thickness, and the underlying tooth‐colored background. Materials with lower translucency and greater thickness are recommended for highly discolored teeth. Vita Mark II was the most suitable material for restorations on darkened substrates.

## 1 Introduction

The ability of dental restorations to mimic the color and optical properties of natural teeth is highly desirable but quite challenging, especially in the anterior region [1, 2]. Computer‐aided design and manufacturing (CAD‐CAM) technologies offer an efficient and well‐recognized method for fabricating dental restorations [3, 4]. This digital workflow has expanded material options, providing a range of compositions and microstructures for producing full‐contour monolithic restorations [3, 5]. Besides traditional monolithic ceramics, new generations of resin matrix ceramic materials were introduced as viable alternatives [6]. Among them, CAD‐CAM resin‐based composites (RBCs) and polymer‐infiltrated ceramic network (PICN) have become popular choices because they combine the superior esthetics of ceramics with the improved fracture toughness, lower elastic modulus, and enhanced repairability of polymers, yielding reliable outcomes [7, 8].

Achieving an optimal esthetic outcome involves various factors beyond material selection with its intrinsic characteristics, including shade, translucency, surface properties, restoration thickness, and underlying substrate color [2, 3, 9, 10, 11, 12, 13]. Understanding how these factors influence the final color of restorations helps us manipulate them to create indirect restorations that mimic the appearance of natural teeth [11]. Dental preparations vary based on clinical indications, ranging from minimally invasive techniques to those involving extensive removal of tooth structure [14]. Modern dentistry emphasizes preserving natural tooth structure whenever possible. For anterior teeth where esthetics are paramount, controlling restoration thickness is essential for achieving a natural appearance. Thicker restorations can affect translucency and color matching with adjacent natural teeth [15]. Therefore, thinner and more translucent materials might be necessary for anterior restorations, especially in non‐discolored teeth.

Esthetic clinical success may be unpredictable when restorative procedures involve masking the color of tooth substrates [16]. Tooth discoloration can stem from endodontic treatment, pulp reactions, metabolic diseases, systemic factors, or extrinsic staining from diet and medications [17]. The choice of material used in restorations may imply the need for a specific thickness to achieve a balance between translucency and opacity for a better natural look [18]. The concept of infinite optical thickness (*X*_∞_) analysis aids in determining the minimum thickness at which a translucent material, when backed by a black background, reaches its maximum light reflectivity and appears nearly opaque [19]. When restoration thickness surpasses the *X*_∞_ value, the material absorbs or scatters all incoming light, eliminating the influence of the background color and displaying its inherent color [13, 20]. However, in situations where the required thickness for covering discolored or darkened teeth is not achievable, clinicians must opt for materials with effective masking abilities [21].

Various methodologies have been employed to evaluate the masking effect of restorative materials, such as the translucency parameter (TP) and contrast ratio (CR) [16, 22]. The use of CIELAB and CIEDE2000 color difference formulas ($∆E_{\mathrm{ab}}^{*}$ and $\Delta E_{00}$) in conjunction with perceptibility and acceptability thresholds (PT and AT) has been recognized as the most appropriate and recommended approach for assessing masking ability [22]. Masking ability is a specific behavioral characteristic of a dental material or structure. Hence, it has been suggested that studies on color differences should focus on evaluating a material or structure over different colored substrates rather than comparing color difference values of different materials/structures [22].

Several studies [2, 11, 12, 17, 21, 23, 24, 25, 26] have investigated the masking ability of CAD‐CAM materials, with only one study [12] considering a combination of various factors. In this study [12], samples of three different thicknesses were evaluated on nine different background shades, although using only vitreous ceramics. Given the widespread use of resin‐matrix materials and feldspathic porcelains in anterior teeth, understanding the interaction of these factors is of both scientific and clinical importance. Additionally, only in this previous study [12], the color coordinates obtained on colored backgrounds were compared with those obtained on a black background, a methodology that is highly relevant for determining the actual influence of the substrate on the final restoration color.

A study [27] was conducted to evaluate the relative masking power of dental porcelain opaque by using dental porcelain glaze as a diluent. The qualitative evaluation of the relative masking power of undiluted opaque porcelain was performed by extrapolating quantitative reflectance spectrophotometry data obtained from diluted opaque porcelains, utilizing the scattering and absorption coefficients (S and K) in the Kubelka–Munk equation. Infinite optical thickness (*X*_∞_) of each specimen was calculated from these coefficients. To the best of the authors' knowledge, no analysis of infinite optical thickness has been conducted to assess the masking ability in CAD‐CAM restoratives to date.

As the masking ability of CAD‐CAM restorative materials across a spectrum of relevant colored substrates and different thicknesses is critical for acceptable esthetic restorations on discolored teeth, this study aimed to evaluate color differences (Δ*E*_00_), relative translucency parameter differences (ΔRTP_00_), and infinite optical thickness (*X*_∞_) to assess the ability of CAD‐CAM resin composites, polymer‐infiltrated ceramic network, and feldspar porcelain at different thicknesses for masking underlying tooth‐colored backgrounds. The null hypotheses tested were that: (H0_1_) CAD‐CAM material composition would not influence the masking ability; (H0_2_) color and translucency differences would not be affected by varying material thickness; and (H0_3_) there would be no difference in the spectral behavior and values of the infinite optical thickness among CAD‐CAM materials.

## 2 Materials and Methods

### 2.1 Specimen Preparation

Four high‐translucency (HT) CAD‐CAM materials (Table 1) in shade A2 were analyzed: Lava Ultimate (LU), Grandio Blocs (GB), VITA Enamic (VE), and VITA Mark II (VM); and nine shades of laboratory composite die material (IPS Natural Die Material system; Ivoclar Vivadent AG, Schaan, Liechtenstein, Germany) were chosen as the colored backgrounds. The sample size calculation with a software program (G*Power v.3.1.9.7; Heinrich‐Heine‐Universität Düsseldorf) and a pilot study determined a minimum of 3 specimens per group at *α* = 0.05 and power = 80%, totaling nine specimens per material. The CAD‐CAM blocks were milled into 7.5 mm diameter cylinders and sectioned into disk samples of three thicknesses (*n* = 3): 0.5, 1.0, and 1.5 mm using a precision saw (IsoMet 1000, Buehler, Lake Bluff, IL, USA). Polishing was performed using 600, 1200, 1500, and 2000 grit SiC carbide papers on a rotary polishing machine (Aropol 2 V; Arotec, São Paulo, SP, Brazil) under water irrigation. The final specimen thickness was verified with a digital caliper (Mitutoyo Digimatic Caliper 150 mm, Mitutoyo, Kawasaki, Japan), and the error was controlled within ±0.1 mm. All specimens were ultrasonically cleaned in distilled water for 20 min, dried with absorbent paper, and stored at 37°C for 24 h before measurements.

**TABLE 1:** CAD‐CAM materials and composite die material evaluated.

Classification	Materials and codes	Manufacturers	Compositions (% in weight)^a^
RBCs^b^	Lava Ultimate HT^d^ A2 (LU)	3 M ESPE	Agglomerated nanoparticles of silica and zirconia (80%), highly cross‐linked polymer matrix composed of Bis‐GMA, UDMA, Bis‐EMA, and TEGDMA (20%). Particle sizes: 20‐nm silica; 4‐ to 11‐nm zirconia
	Grandio Blocs HT^d^ A2 (GB)	VOCO	Nanohybrid filler particles of nanosilica and barium glass (86%) dispersed in an organic polymeric matrix composed of UDMA and DMA (14%)
PICN^c^	VITA Enamic HT^d^ A2 (VE)	VITA Zahnfabrik	Polymer‐infiltrated feldspathic ceramic network (86% ceramic), resin polymer composed of UDMA and TEGDMA (14%)
Feldspar porcelain	VITA Mark II HT^d^ A2 (VM)	VITA Zahnfabrik	SiO_2_ (56%–64%), Al_2_O_3_ (20%–23%), Na_2_O (6%–9.0%), K_2_O (6%–8.0%), CaO (0.3–0.6%), TiO_2_ (0%–0.1%)
Light‐curing shaded composite die	IPS Natural Die Material (ND)	Ivoclar Vivadent AG	Paste of polyesterurethanedimethacrylate, silicon dioxide, paraffin oil, copolymer, initiators, stabilizers, and pigments

^a^ Information given by the respective manufacturers.

^b^ Resin‐based composites.

^c^ Polymer‐infiltrated ceramic network.

^d^ High‐translucency level.

**FIGURE 1:**
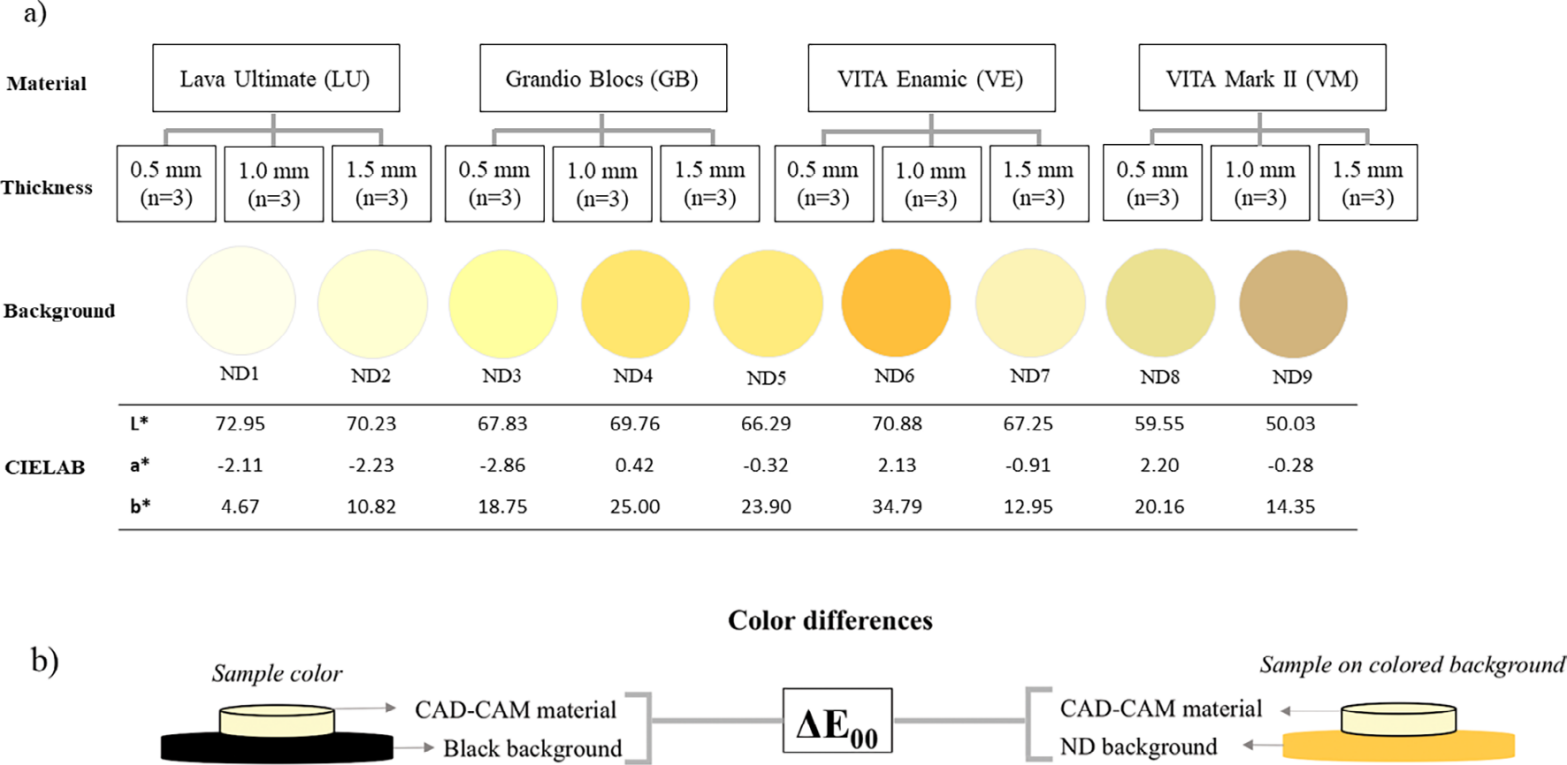
(a) Schematic representation of group division according to thicknesses, background color coordinates, and the experimental design; (b) color difference calculations.

Disk‐shaped colored backgrounds (*n* = 1/shade; 18.0 × 1.0 ± 0.1 mm) were fabricated from nine shades (ND1, ND2, ND3, ND4, ND5, ND6, ND7, ND8, and ND9) of the IPS Natural Die Material system to simulate different colors of abutment teeth. Each resin‐based composite shade was applied to a custom‐designed metallic matrix mold between two overlapping glass plates. Light‐polymerization was performed using a light‐curing unit (VALO Grand, Ultradent Products, South Jordan, USA) with an average irradiance of 1000 mW/cm^2^ for 40 s on each side [28]. The disks were manually polished with SiC carbide papers (1200 and 1500 grit) under water irrigation for 1 min on both sides, cleaned with distilled water, and dried with compressed air. The experimental design, group distribution, and color coordinates of the backgrounds are shown in Figure 1a.

### 2.2 Spectral Reflectance Measurement

The spectral reflectance of the colored backgrounds and materials at different thicknesses was measured using a non‐contact setup comprised of a calibrated spectroradiometer (SpectraScan PR 670, Photo Research, Chatsworth, CA, USA), a xenon arc lamp (300 W, Newport Stratford, Franklin, MA, USA), and two fiber‐optic light cables (Model 70050, Newport Stratford, Franklin, MA, USA) positioned on a custom‐made optical table [29]. The spectroradiometer was placed 40 cm away from the sample surface with a 45°/0° illuminating/measuring geometry [29, 30]. The materials were measured (*n* = 3) against standard white (L* = 94.2, a* = 1.3 and b* = 1.7) and black (L* = 3.1, a* = 0.7 and b* = 2.4) backgrounds (50 × 50 mm ceramic tiles, Ceram, Staffordshire, UK), and over the nine shades of ND backgrounds in a completely dark room. Spectral reflectance values were converted into CIE L* a* b* color coordinates using the CIE 2° Standard Observer and CIE D65 Standard Illuminant, and then chroma (C*) and hue angle (*h°*) coordinates were calculated.

### 2.3 Color Difference and Relative Translucency Parameter

Computations were performed for CIEDE2000 color differences (Δ*E*_00_) between the sample on each colored background and against the black background for the same material and thickness (Figure 1b). Color differences between different thicknesses of a material against the same colored background were also determined using the equation [31]:

$$∆E_{00}={\left[{\left(\frac{∆L^{\prime }}{K_{L}S_{L}}\right)}^{2}+{\left(\frac{∆C^{\prime }}{K_{C}K_{C}}\right)}^{2}+{\left(\frac{∆H^{\prime }}{K_{H}S_{H}}\right)}^{2}+R_{T}\left(\frac{∆C^{\prime }}{K_{C}S_{C}}\right)\left(\frac{∆H^{\prime }}{K_{H}S_{H}}\right)\right]}^{1/2}$$

where Δ*L*ʹ, Δ*C*ʹ, and Δ*H*ʹ represent the differences in lightness, chroma, and hue. *R*_T_ is the rotation function that accounts for the interaction between chroma and hue differences in the blue region of color space. The weighting functions *S*_L_, *S*_C_, and *S*_H_ adjust the total color difference for variation in the location of the color difference pair in *L*ʹ, *a*ʹ, and *b*ʹ coordinates, and the parametric factors *K*_L_, *K*_C_, and *K*_H_ are correction terms for experimental conditions, considered to be 1 in this study.

Color differences between different thicknesses were evaluated through comparisons with the 50%:50% perceptibility and acceptability (PT_00_ = 0.8 and AT_00_ = 1.8 Δ*E*_00_ units) color thresholds [32].

Relative translucency parameter (RTP_00_) values were determined by calculating Δ*E*_00_ between the color coordinates measured over black and white backgrounds, following the equation [31, 33]:

RTP00=LB′−LW′kLSL2+CB′−CW′kCSC2+HB′−HW′kHSH2+RTCB′−CW′kCSCHB′−HW′kHSH12

where the subscripts “B” and “W” refer to the lightness (*L*′), chroma (*C*′), and hue (*H*′) of the specimens over the black and the white backgrounds, respectively.

The relative translucency parameter difference (ΔRTP_00_) was assessed between CAD‐CAM materials at the same thickness and among different thicknesses for each material by the formula as follows:

$$\Delta {\mathrm{RTP}}_{00}={\mathrm{RTP}}_{002}-{\mathrm{RTP}}_{001}$$

Translucency differences were evaluated using published data of 50%:50% translucency difference perceptibility (TPT_00_ = 0.62) and difference acceptability (TAT_00_ = 2.62) thresholds [33].

### 2.4 Infinite Optical Thickness

The infinite optical thickness (*X*_∞_) was calculated algebraically, as follows [19]:

X∞=1bSArctgh1−0.999aRI0.999bRI

Secondary optical constants (*a* and *b*), light reflectivity (RI), and scattering coefficient (S) were calculated from reflectance data of the specimen as a function of wavelength at every 2.0 nm using Kubelka–Munk equations [34, 35].

The root mean square error (RMSE) and the goodness‐of‐fit coefficient (GFC) metrics were used to evaluate the level of similarity in spectral behavior and values of *X*_∞_ among materials [36, 37]. RMSE evaluates the absolute differences between two spectral signals, focusing on the magnitude of the difference, and is not independent of scale factors. An RMSE = 0 indicates a perfect match, while an RMSE of around 2.0% indicates a good comparison of metrics spectral quality [37]. The GFC is a metric that represents the cosine of the angle formed between two samples compared in the high‐dimensional vector space of spectral curves. A GFC = 1 indicates a perfect match. Values of GFC greater than or equal to 0.999 and 0.9999 correspond to good and excellent spectral matches, respectively.

### 2.5 Statistical Analysis

The Δ*E*_00_ data were analyzed for normality and variance homogeneity. The values did not follow the assumption of normality and were therefore analyzed using non‐parametric statistical tests. The comparison among materials for each thickness on the same background was performed using the Kruskal–Wallis test, followed by the Mann–Whitney test for pairwise comparisons. The significance level was set at *α* = 0.05. IBM SPSS v23.0 for Windows (IBM Corp, Armonk, NY, USA) was used for the statistical analyses.

## 3 Results

Δ*E*_00_ values for the CAD‐CAM materials at different thicknesses upon the nine colored backgrounds, with the coordinates being compared with those obtained on the black background, are presented in Table 2. The feldspar porcelain (VM) consistently exhibited the lowest Δ*E*_00_ values among the CAD‐CAM materials across all thicknesses and backgrounds (*p* < 0.001). With few exceptions, for 0.5 mm and 1.0 mm, LU and GB showed no significant differences, while for 1.5 mm, LU had lower Δ*E*_00_ values than GB (*p* ≤ 0.005). In general, LU showed lower Δ*E*_00_ values than VE for 0.5 mm (*p* ≤ 0.005), but the opposite behavior was observed for 1.0 mm samples (*p* = 0.001), and no difference was verified between both materials for 1.5 mm. Except for 1.0 mm against ND6 and ND7, GB and VE did not differ, regardless of the thickness.

**TABLE 2:** Mean and ± standard deviations of Δ*E*_00_ between the sample color and the sample on tooth‐colored backgrounds.

Background	Thickness	Material			
		LU	GB	VE	VM
ND1	0.5 mm	16.19 ± 0.34^B^	16.89 ± 1.08^BC^	17.41 ± 0.17^C^	10.16 ± 0.11^A^
	1.0 mm	10.37 ± 0.28^B^	10.41 ± 0.29^B^	10.11 ± 0.18^B^	8.14 ± 0.15^A^
	1.5 mm	7.77 ± 0.07^B^	8.23 ± 0.09^C^	8.29 ± 0.30^C^	5.90 ± 0.19^A^
ND2	0.5 mm	15.75 ± 0.33^B^	16.67 ± 1.07^BC^	16.65 ± 0.28^C^	9.90 ± 0.14^A^
	1.0 mm	9.97 ± 0.29^B^	10.08 ± 0.33^B^	9.72 ± 0.14^B^	7.74 ± 0.11^A^
	1.5 mm	7.48 ± 0.07^B^	7.87 ± 0.13^C^	8.26 ± 0.67^BC^	5.50 ± 0.21^A^
ND3	0.5 mm	15.56 ± 0.32^B^	16.50 ± 1.06^C^	16.18 ± 0.52^BC^	9.91 ± 0.21^A^
	1.0 mm	10.13 ± 0.20^C^	10.08 ± 0.40^BC^	9.69 ± 0.24^B^	7.76 ± 0.13^A^
	1.5 mm	7.38 ± 0.13^B^	7.67 ± 0.03^C^	7.84 ± 0.66^BC^	5.50 ± 0.19^A^
ND4	0.5 mm	17.26 ± 0.37^B^	18.30 ± 1.05^C^	17.98 ± 0.36^C^	11.01 ± 0.03^A^
	1.0 mm	11.19 ± 0.20^C^	11.27 ± 0.38^BC^	10.79 ± 0.13^B^	8.57 ± 0.10^A^
	1.5 mm	8.40 ± 0.17^B^	8.56 ± 0.19^B^	8.75 ± 0.34^B^	6.12 ± 0.23^A^
ND5	0.5 mm	15.44 ± 0.34^B^	16.33 ± 0.95^C^	16.35 ± 0.28^C^	9.89 ± 0.07^A^
	1.0 mm	9.82 ± 0.27^C^	9.88 ± 0.37^BC^	9.39 ± 0.20^B^	7.72 ± 0.15^A^
	1.5 mm	7.49 ± 0.11^B^	7.83 ± 0.19^C^	7.49 ± 0.52^C^	5.48 ± 0.11^A^
ND6	0.5 mm	19.36 ± 0.36^B^	20.23 ± 0.88^C^	19.90 ± 0.29^C^	12.53 ± 0.11^A^
	1.0 mm	12.92 ± 0.02^C^	12.73 ± 0.37^C^	12.16 ± 0.20^B^	9.92 ± 0.15^A^
	1.5 mm	9.45 ± 0.14^B^	9.69 ± 0.16^C^	9.48 ± 0.38^BC^	6.92 ± 0.14^A^
ND7	0.5 mm	14.17 ± 0.30^B^	14.94 ± 1.07^BC^	14.76 ± 0.29^C^	8.67 ± 0.03^A^
	1.0 mm	9.18 ± 0.05^C^	9.19 ± 0.32^C^	8.80 ± 0.24^B^	6.93 ± 0.14^A^
	1.5 mm	6.64 ± 0.09^B^	7.00 ± 0.30^C^	7.42 ± 0.61^BC^	4.79 ± 0.12^A^
ND8	0.5 mm	12.36 ± 0.28^B^	13.20 ± 1.02^B^	12.63 ± 0.25^B^	7.56 ± 0.11^A^
	1.0 mm	8.23 ± 0.17^C^	8.06 ± 0.49^BC^	7.72 ± 0.12^B^	6.14 ± 0.08^A^
	1.5 mm	5.99 ± 0.11^B^	6.17 ± 0.22^B^	6.60 ± 0.63^B^	4.25 ± 0.15^A^
ND9	0.5 mm	7.74 ± 0.26^B^	8.19 ± 0.99^BC^	8.37 ± 0.26^C^	4.50 ± 0.02^A^
	1.0 mm	5.04 ± 0.22^C^	4.87 ± 0.41^BC^	4.54 ± 0.18^B^	3.79 ± 0.15^A^
	1.5 mm	3.56 ± 0.09^B^	3.77 ± 0.36^B^	3.87 ± 0.74^B^	2.45 ± 0.11^A^

*Note:* Different superscript letters in lines indicate significant differences (*p* < 0.05) among materials at the same thickness.

**FIGURE 2:**
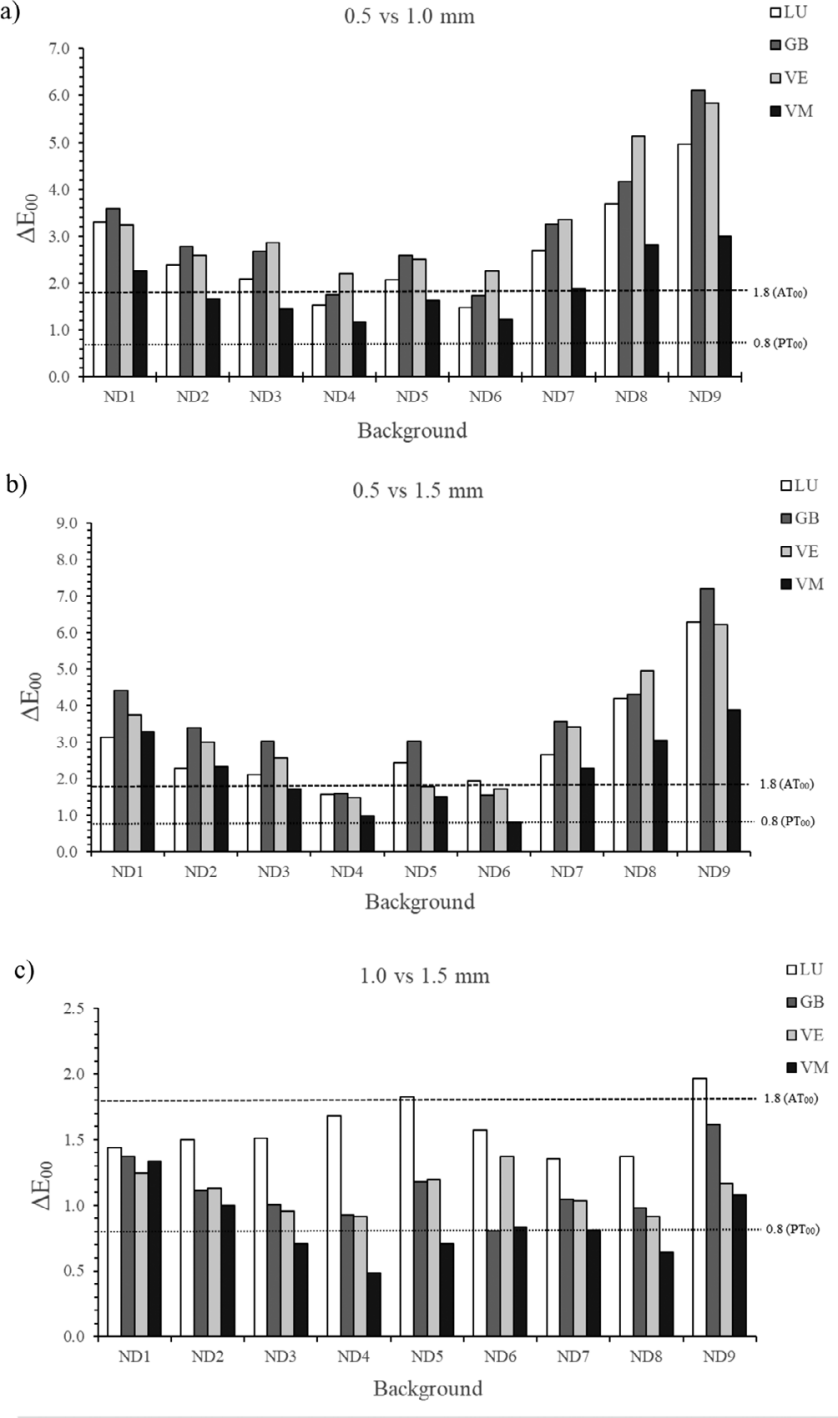
Color differences (Δ*E*_00_) between different thicknesses for each material and background and threshold interpretation ratings. (a) Δ*E*_00_ values of 0.5 vs. 1.0 mm, (b) Δ*E*_00_ values of 0.5 vs. 1.5 mm; and (c) Δ*E*_00_ values of 1.0 vs. 1.5 mm.

Figure 2 illustrates the Δ*E*_00_ values between different thicknesses for each material and background compared to PT_00_ and AT_00_ color thresholds. ND9 produced the highest color differences at various thicknesses for all materials, while VM showed lower differences across various thicknesses and colored backgrounds. Less frequently in the intermediate backgrounds, ND4, ND5, and ND6, the Δ*E*_00_ values between 0.5 mm and 1.5 mm (Figure 2b) were generally higher than 0.5 mm–1.0 mm (Figure 2a). The Δ*E*_00_ values exceeded AT_00_ between 0.5 mm–1.0 mm (Figure 2a) and 0.5 mm–1.5 mm (Figure 2b), with the exceptions more concentrated in ND4 and ND6. Only LU exhibited color differences higher than AT_00_ between 1 mm–1.5 mm samples for ND5 and ND9 (Figure 2c). VM was the only material with imperceptible color differences between 1.0 mm–1.5 mm (Figure 2c), but only for ND3, ND4, ND5, ND7, and ND8.

**FIGURE 3:**
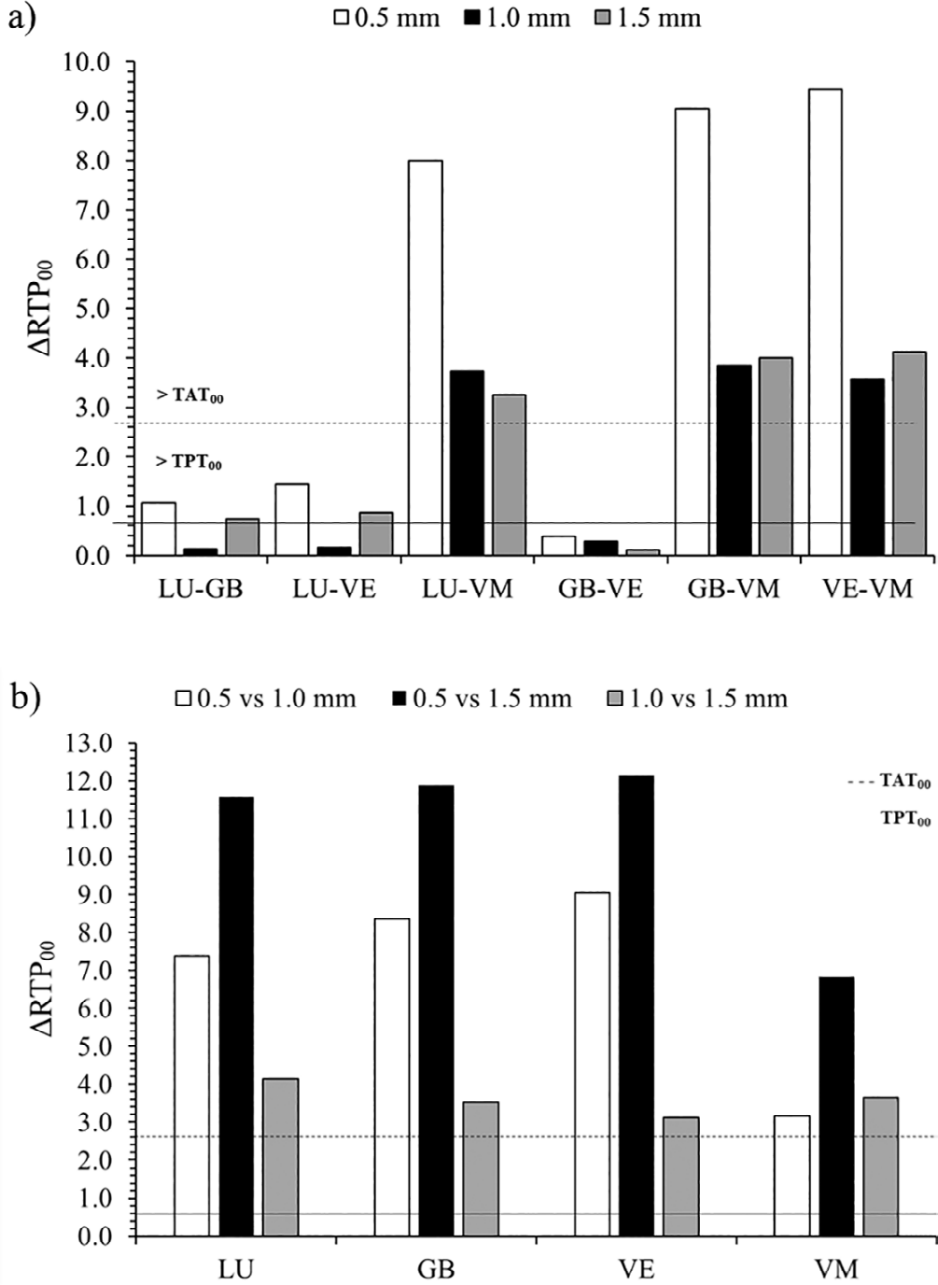
Mean relative translucency parameter differences (ΔRTP_00_) (a) between CAD‐CAM materials at the same thickness; and (b) between sample thicknesses of a CAD‐CAM material. TPT_00_, translucency difference perceptibility threshold; TAT_00_, translucency difference acceptability threshold.

The ΔRTP_00_ values between the CAD‐CAM materials within each thickness and at the different thicknesses within each material, as well as the TPT_00_ and TAT_00_ translucency thresholds, are shown in Figure 3a,b, respectively. VM exhibited a translucency difference higher than TAT_00_ with LU, GB, and VE at the three thicknesses. However, the ΔRTP_00_ values between LU, GB, and VE were generally within acceptable ranges. Only GB‐VE at all thicknesses, and LU‐GB and LU‐VE at 1.0 mm thickness, showed imperceptible translucency differences. All materials reported ΔRTP_00_ values higher than TAT_00_ when comparing different thicknesses (Figure 3b).

**FIGURE 4:**
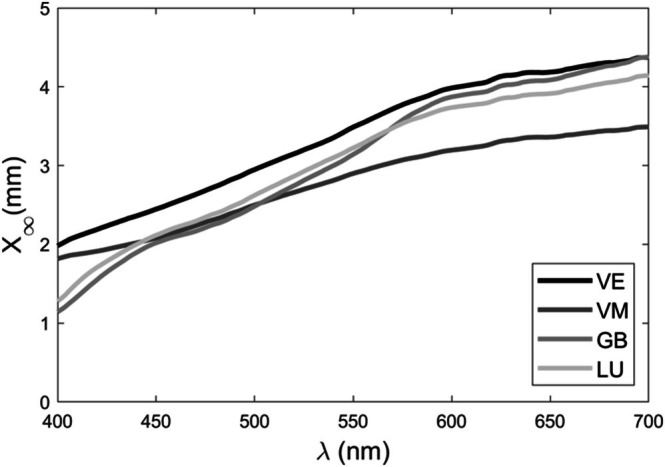
Spectral distribution of infinite optical thickness (*X*_∞_) of the CAD‐CAM materials.

The infinite optical thickness (*X*_**∞**_) values were wavelength‐dependent, increasing as the wavelength increased, irrespective of material (Figure 4). The CAD‐CAM materials were more opaque at lower wavelengths than at higher wavelengths, with the effect being less pronounced for VM. The *X*_**∞**_ values ranged from 1.28 to 4.14 mm for LU, 1.14 to 4.38 mm for GB, 1.98 to 4.37 mm for VE, and 1.82 to 3.49 mm for VM. When comparing the materials, a RMSE below 2.0% and a GFC ≥ 0.999 for LU‐GB indicated a good spectral match and good comparative spectral values for these resin‐based materials for *X*_**∞**_ (Table 3). VE‐VM reported a GFC ≥ 0.999 and RMSE above 2.0%, suggesting a good spectral match but not good comparative spectral values (Table 3).

**TABLE 3:** RMSE and GFC metrics for infinite optical thickness (*X*_∞_) between evaluated CAD‐CAM materials at 1.5 mm.

Performance metric	LU‐GB	LU‐VE	LU‐VM	GB‐VE	GB‐VM	VE‐VM
RMSE	0.24255	0.54492	0.93334	0.62433	1.13631	1.05174
GFC	0.99946	0.99805	0.99388	0.99627	0.99060	0.99906

## 4 Discussion

The complex interaction of underlying structure, restoration thickness, and composition of monolithic restorative materials makes color reproduction one of the greatest clinical challenges [8, 16]. This study focused on contributing to existing knowledge by evaluating the masking ability of widely used CAD‐CAM materials at different thicknesses through the replication of tooth abutments in various shades to address the importance of effective masking for achieving predictable and esthetically pleasing outcomes in esthetic dentistry.

The results revealed significant color differences among the materials studied, regardless of thickness and background, leading to the rejection of the first hypothesis. Since comparisons between different thicknesses produced color and translucency difference values above the PT_00_ and TPT_00_ thresholds, the second hypothesis was rejected. Except for LU‐GB and VE‐VM, the spectral analysis indicated differences in comparative spectral quality metrics and spectral behavior between the *X*_∞_ values of the materials evaluated. Therefore, the third hypothesis was also rejected.

Previous studies have calculated color changes by comparing samples measured against a pair of natural dye background shades to assess the masking effect of dental materials [16, 38]. However, this approach does not directly indicate which background had a greater impact on the color change. On the other hand, in this study, the authors aimed to assess the color coordinates of samples on a black background and calculate the color differences between the sample color and the sample against each ND tooth‐colored background, a method described in a previous study [12].

Among the materials tested, feldspar porcelain showed the lowest Δ*E*_00_ and ΔRTP_00_ values across three thicknesses and against all colored backgrounds. Translucency was described as a key first factor influencing the masking effect of restorative materials by affecting light transmission and reflection [33, 39]. Higher translucency reduces the ability to mask underlying colors [39, 40]. The superior masking performance of VM can be attributed to its lower translucency compared to LU, GB, and VE materials [41]. Relative translucency parameter differences data for VM confirmed this behavior. Comparisons between VM and other CAD‐CAM materials revealed the highest ΔRTP_00_ values for all thicknesses, exceeding the acceptable limit. Additionally, VM exhibited the lowest ΔRTP_00_ values across different sample thicknesses, aligning with its lowest Δ*E*_00_ values and indicating less influence of backgrounds on the VM material color.

A recent study [41] using the same materials found that VM exhibited the highest scattering and absorption, which determine how light interacts with dental materials. Materials with high scattering diffuse light within the structure, and this diffusion reduces the contrast between the restoration and the underlying substructure by distributing light more evenly [39]. As a result, the material can effectively cover dark tooth abutments, enhancing its masking ability. The absorption coefficient also contributes to masking by preventing light from penetrating deeply to reveal the background color. These findings probably explain why VM had less influence on the substrate color. Thickness plays a crucial role in enhancing these effects, as thicker material increases both scattering and absorption pathways. This may be related to reduced color change with increasing thickness, not only in VM but also in other materials. In contrast, one study reported lower masking ability in Vita Mark II compared to Lava Ultimate and Vita Enamic, regardless of thickness and substrate evaluated [25]. This difference in behavior may be due to the use of different substrates in the two studies or variations in the methodology used to calculate color differences.

When comparing LU and GB, the statistical similarity observed for 0.5 and 1.0 mm can be attributed to their comparable translucency characteristics, as demonstrated in the current investigation and by one previous study [41]. The slight difference in translucency between these two materials was found to be below the perceptibility threshold. Following this line of reasoning, a similar behavior was expected for 1.5 mm, but the findings did not align with this expectation, and we were unable to find an explanation. No previous studies comparing these materials in the same context as the present study were identified.

Studies on the color change between LU and VE with a 0.5 mm thickness were not found. Regarding the 1.0 mm thickness, two previous studies [24, 25] supported the lower color change of VE compared to LU. This difference is possibly due to the lower translucency of VE, which offers a more effective masking effect than LU. These materials also exhibit different spectral behaviors in terms of scattering, absorption, transmittance, and light reflectivity [41], which could explain the finding. When considering the thickness of 1.5 mm, two studies [24, 25] have demonstrated a lower color change in VE compared to LU, which contrasts with the statistical equality found in the present study. The reason for this difference in behavior between the studies is unclear, but the current study indicates that with thicker samples, both materials demonstrate a similar ability to mask the same background.

In a clinical setting, adjusting the thickness of a restoration can alter the translucency and opacity ratio [42, 43], resulting in color differences that may be deemed imperceptible, acceptable, or unacceptable. The analysis of Δ*E*_00_ values between thicknesses based on PT_00_ and AT_00_ thresholds generally revealed unacceptable color differences when the thinner thickness (0.5 mm) was considered against ND7, ND8, and ND9 darker backgrounds. The masking effectiveness of indirect restorations on discolored teeth tends to be more challenging, as they absorb more light and reflect less, making their color influence more critical in the final appearance of restorations. Comparing 1.0 to 1.5 mm resulted in smaller color differences for all materials, with values generally within acceptable limits against all backgrounds. Consistent with previous studies [2, 11, 12, 23, 24, 44], increasing the thickness improved the color masking ability of the materials by reducing translucency. In this comparison, VM was the only material that exhibited imperceptible color changes, possibly related to its lower translucency compared to the other materials at all three thicknesses. Thus, it is suggested that changes in thickness had a less significant impact on the color of VM.

For discolored teeth, restorative materials may require a specific thickness to achieve a balance between translucency and opacity for a more natural appearance. The infinite optical thickness (*X*_∞_) is the minimum thickness at which a translucent material, when placed against a black background, will attain its maximum light reflectivity and become nearly opaque [19]. When the thickness of a restoration exceeds the *X*_∞_, the influence of the background is eliminated [20, 41]. Understanding this concept is crucial for light interaction, guiding material selection, and determining the minimum thickness required for optical masking while considering other clinical factors such as strength and translucency. Dental restorations like veneers, with lower *X*_∞_ values, allow some light transmission, replicating the natural translucency of teeth, which is important in the anterior region. The study revealed that the 1.5 mm thick LU and GB samples exceeded the *X*_∞_ within the 400–420 nm spectrum, indicating reduced background influence at shorter wavelengths, potentially contributing to their lower Δ*E*_00_ values at this thickness. The fact that VE and VM at 1.5 mm thickness did not reach their *X*_∞_ at all wavelengths (see Figure 4) suggests that this thickness may still be insufficient to completely mask the underlying substrate, as reflected in the Δ*E*_00_ values.

Except for shorter wavelengths, none of the high‐translucency CAD‐CAM materials evaluated achieved infinite optical thickness at 1.5 mm, indicating the need for a thickness greater than 1.5 mm to achieve complete background masking. LU and GB exhibited a good spectral match and comparable spectral values for infinite optical thickness, suggesting that both materials can effectively eliminate background influence above 1.5 mm with consistent variations in restoration thickness. This behavior does not apply to VE‐VM, as no spectral matches were found for their *X*_∞_ values. Regarding VM, it demonstrated the best ability to mask backgrounds across all three thicknesses and would reach infinite optical thickness at a lower thickness compared to the other materials. While surpassing *X*_∞_ ensures a complete masking effect [19, 20, 41], the results showed that the materials can produce acceptable color differences at specific thicknesses, making them suitable for restorative procedures. This is particularly beneficial in cases where tooth structure preservation and anatomical constraints restrict the desired thickness.

Our study protocol provided detailed information on the masking ability of four different CAD‐CAM materials, particularly in challenging clinical situations involving darker substrates. The results indicate that certain CAD‐CAM restorative materials may exhibit adequate masking capacity even over discolored and darker backgrounds, provided they are used at increased thicknesses, especially in monolithic restorations. This finding is clinically significant for achieving esthetically pleasing outcomes when concealing the substrate is necessary. When dealing with lighter or moderately discolored teeth, their impact on the final color of a restoration remains significant. However, in these cases, it is possible and becomes advantageous to use materials with higher translucency, as it allows the underlying tooth structure to contribute positively to the overall esthetic. This approach promotes more biomimetic and natural‐looking restorations. Therefore, while masking is critical in darker scenarios, translucency can be beneficial in more favorable clinical situations.

The main limitation of this study was the lack of consideration for the cementation of CAD‐CAM material samples on the composite die substrate, which may have influenced the results. However, a recent study found that the cement shade had the least influence on the final color of resin‐matrix ceramics samples compared to other factors [26]. This study utilized a spectroradiometric measurement set‐up to obtain accurate non‐contact readings that closely resemble the viewing conditions of human visual assessments [45]. Furthermore, all available shades of the composite die material were used to simulate various tooth‐colored abutments encountered in clinical scenarios. The comprehensive analysis of color differences, translucency, and particularly data on infinite optical thickness enhances the significance of this work and fills an important gap in the field.

## 5 Conclusions

Within the limitations of this study and based on the findings, the following conclusions were drawn. The masking ability of the high‐translucency CAD‐CAM materials varied depending on their composition, thickness, and the color of the underlying background. Among the materials tested, the feldspar porcelain Vita Mark II exhibited a superior masking effect at all three thicknesses, making it a suitable material for restorations involving darkened substrates. The infinite optical thickness indicated that it is recommended to use the CAD/CAM materials with a thickness greater than 1.5 mm to ensure complete elimination of background influence across all wavelengths.

## Conflicts of Interest

The authors declare no conflicts of interest.

## Data Availability

Research data are not shared.
